# Nuciferine Ameliorates Lipotoxicity-Mediated Myocardial Ischemia–Reperfusion Injury by Reducing Reverse Electron Transfer Mediated Oxidative Stress

**DOI:** 10.3390/nu18030425

**Published:** 2026-01-27

**Authors:** Man Wang, Xiaobing Shi, Yufeng Zhou, Jianhui Feng, Yining Diao, Gang Li, Zhenhua Wang, Chengjun Ma

**Affiliations:** 1 Center for Mitochondria and Healthy Aging, School of Life Sciences, Yantai University, Yantai 264005, China; wangman202302@163.com (M.W.); m17865562177@163.com (Y.Z.); jhfeng0122@163.com (J.F.); ligang@ytu.edu.cn (G.L.); 2 Xinjiang Production and Construction Corps Key Laboratory of Protection and Utilization of Biological Resources in Tarim Basin, College of Life Science, Tarim University, Alar 843300, China; 15966463268@163.com (X.S.); addiaoyining@163.com (Y.D.)

**Keywords:** nuciferine, myocardial ischemia–reperfusion injury, mitochondrial reverse electron transport, Sirt1

## Abstract

**Background/Objectives**: The widespread adoption of high-fat diets has contributed to a rising incidence of metabolic disorders and associated cardiovascular diseases. This trend exacerbates myocardial ischemia–reperfusion (I/R) injury following interventional or thrombolytic therapy for acute myocardial infarction, leading to higher mortality and heart failure in affected individuals with metabolic dysregulation, for whom effective interventions are limited. Nuciferine, which possesses anti-inflammatory, antioxidant, and metabolic regulatory properties, has shown potential in improving post-I/R cardiac function, yet its mechanism remains unclear. **Methods**: This study utilized an ex vivo mouse heart model perfused with high-glucose/high-fatty acid solutions to establish a metabolic stress condition mimicking key aspects of the diabetic milieu and to evaluate the underlying mechanisms of nuciferine. Complementarily, a model of lipotoxicity combined with hypoxia/reoxygenation (H/R) injury was established in human cardiomyocyte cells (AC16). **Results**: Nuciferine significantly improved post-I/R functional recovery and attenuated succinate accumulation, an effect comparable to the succinate dehydrogenase (SDH) inhibitor dimethyl malonate (DMM). Mechanistically, nuciferine bound to an SDH subunit, inhibiting its activity and subsequent reactive oxygen species (ROS) production via mitochondrial reverse electron transport (RET). It also activated Sirt1-dependent pathways, mitigating apoptosis and mitochondrial dysfunction in AC16 cardiomyocytes. The Sirtuin 1 (Sirt1) inhibitor selisistat (EX527) abolished nuciferine’s protection, while DMM mirrored its efficacy, underscoring nuciferine’s dual role in inhibiting SDH-mediated RET and activating Sirt1 in alleviating I/R injury under metabolic stress conditions. **Conclusions**: These findings suggest that nuciferine confers cardioprotection by simultaneously attenuating RET-related oxidative stress and activating Sirt1.

## 1. Introduction

The global prevalence of cardiovascular diseases (CVDs) is largely attributed to high-fat diets and sedentary lifestyles, with myocardial infarction being the most common CVD. This disease primarily stems from coronary atherosclerosis, leading to luminal stenosis, which compromises myocardial blood supply [1]. Patients with metabolic disorders face higher mortality rates post-acute myocardial infarction and a greater risk of heart failure. These outcomes are linked to elevated blood glucose and free fatty acids (FFAs), which reduce myocardial stress resistance and worsen ischemia–reperfusion injury [2,3]. Although early blood flow restoration after myocardial ischemia can lessen myocardial damage and infarct size [4], the associated myocardial ischemia/reperfusion (MI/R) injury involves oxidative stress and cell death, which will result in poor prognosis and recurrence [5,6].

Oxidative stress is crucial in myocardial ischemia, inducing cardiomyocyte death via excessive reactive oxygen species (ROS) generation. During myocardial infarction, the burst of ROS generated by reverse electron transport (RET) at mitochondrial complex I is a crucial trigger for myocaridal injury during reperfusion [7]. The process of reverse electron transport occurs when electrons are forced back from the coenzyme Q (CoQ) pool onto the flavin mononucleotide (FMN) site, enabling the reduction of NAD^+^ to NADH [8,9]. Emerging evidence indicates that succinate dehydrogenase (SDH) is a key mediator of RET in ischemia–reperfusion (I/R) injury [10,11]. Ischemia-induced oxygen deprivation causes excessive reduction of the CoQ pool, forcing SDH to utilize the accumulated electrons for fumarate reduction, thereby generating succinate [7]. Metabolomic analyses have confirmed that succinate, along with hypoxanthine and xanthine, accumulates in ischemic cardiac tissue across species (rats, rabbits, pigs, and humans), establishing it as a conserved marker of tissue ischemia [12]. Consequently, a comprehensive analysis of the pathways involved in preventing succinate accumulation in ischemic/hypoxic tissues may be a useful approach to identify novel therapeutic targets.

Nuciferine, an isoquinoline alkaloid from lotus leaves (Nelumbo nucifera), demonstrates significant pharmacological effects, including anti-inflammatory, antioxidant, and glucolipid metabolic regulation [13,14]. Previous studies have demonstrated that nuciferine enhanced the endogenous antioxidants and controlled ROS-mediated apoptosis against doxorubicin-induced cardiotoxicity [15]. Based on our prior investigation, which led to the isolation of four aporphine alkaloids—including nuciferine—from lotus leaf extract using high—speed countercurrent chromatography, we demonstrated their efficacy in improving glucose and lipid metabolic profiles in adipocytes. This metabolic amelioration was found to be mediated through activation of the 5′ AMP-activated protein kinase (AMPK) signaling pathway [16]. A substantial body of evidence corroborates that AMPK activation augments NAD^+^ levels, subsequently enhancing the deacetylase activity of Sirtuin 1 (Sirt1) and facilitating the deacetylation of its downstream substrates [17]. As a NAD^+^-dependent deacetylase, Sirt1 is a master regulator of metabolic homeostasis and mitochondrial function [18]. However, its mode of action under metabolic disorder conditions induced by RET still lacks systematic elaboration. It is well-documented that RET triggers NAD^+^/NADH imbalance, thereby suppressing the pro-longevity sirtuin/FOXO/autophagy pathway [19]. The NAD^+^/sirtuin signaling pathway counteracts this by activating mitochondrial stress responses and maintaining functional integrity, thereby offering a potential mechanism to inhibit RET-related mitochondrial diseases [20,21]. Emerging as a critical regulator of myocardial I/R injury, Sirt1 has been shown to mediate key protective responses [22]. Accumulating evidence indicates that Sirt1 activation protects against I/R injury through multiple mechanisms, such as attenuating inflammatory responses and apoptosis, maintaining mitochondrial integrity, and alleviating oxidative stress. Notably, the antioxidant properties of Sirt1 are achieved partly via upregulation of major antioxidant enzymes and suppression of ROS generation [23,24]. However, the ability of nuciferine to mitigate myocardial I/R injury and the underlying molecular mechanisms, particularly concerning mitochondrial bioenergetics, metabolic regulation, and associated signaling pathways, remain unclear to date. To address these questions, we employed an integrated multi-level experimental strategy. First, an MI/R injury model using isolated mouse hearts was established to evaluate functional recovery. In parallel, AC16 human cardiomyocyte cell line under hypoxia/reoxygenation (H/R) injury conditions was used to examine mitochondrial responses, including bioenergetics and oxidative stress. Furthermore, molecular docking and dynamics simulations were performed to characterize the binding mode and affinity of nuciferine toward key mitochondrial targets. Together, these complementary approaches provide systematic evidence that nuciferine protects the heart through the regulation of mitochondrial function and signaling pathways.

## 2. Materials and Methods

### 2.1. Animals

Male ICR mice (8 weeks old) were purchased from Jinan Pengyue Experimental Animal Breeding Co., Ltd. (Jinan, China) (production license: SCXK(Lu) 2022 0006). They were maintained under controlled temperature conditions (23 ± 1 °C) with a standard 12 h light/dark cycle, and fed with normal chow and ad libitum with free access to water. The animal study protocol was approved by the Ethics Committee of Yantai University (Registration no.: YDLL2024M368; approved on 21 October 2024).

### 2.2. Study Design and Experimental Protocols

**Figure 1:**
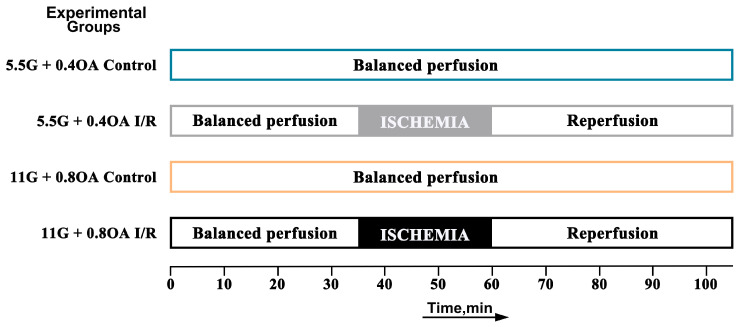
Experimental design and flowchart for investigating the effect of high glucose (G) and oleic acid (OA) on exacerbating myocardial ischemia–reperfusion injury: normal glucose/oleic acid system (5.5 G + 0.4 OA) and high glucose/oleic acid system (11 G + 0.8 OA).

To establish an MI/R injury model exacerbated by high glucose and oleic acid, mice were divided into four groups (*n* = 6 each): a normal group under a conventional system, and an I/R group; a normal group under a high glucose and oleic acid system, and an I/R group, with 6 mice in each group (Figure 1).

**Figure 2:**
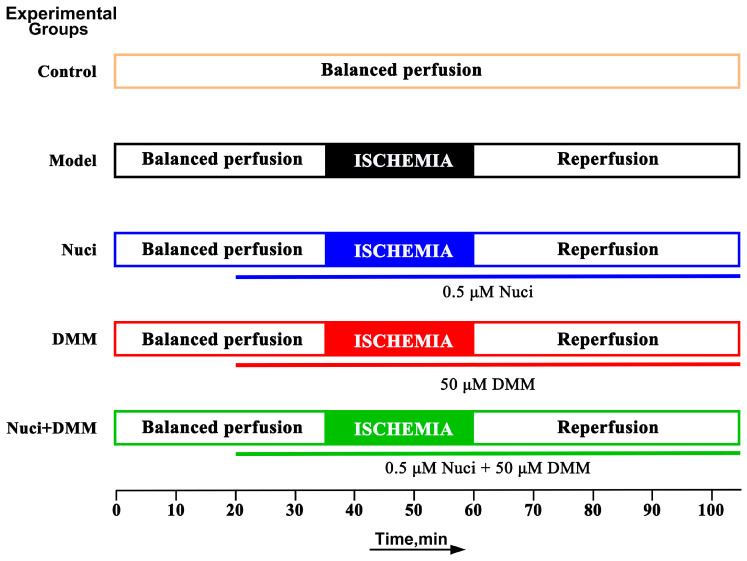
Experimental design and flowchart for investigating the protective effect of nuciferine (Nuci) and dimethyl malonate (DMM) on high glucose (G) and oleic acid (OA) aggravated myocardial ischemia–reperfusion injury.

To evaluate the cardioprotective effects of nuciferine, mice were randomly assigned to five groups (*n* = 6 each): Control, I/R, 0.5 µM Nuci + I/R, 50 µM DMM + I/R, and DMM + Nuci + I/R. Prior to ischemia, hearts were equilibrated with normal KH buffer for 20 min, followed by 15 min of perfusion with drug-containing solution. Ischemia was induced for 25 min, followed by 45 min of reperfusion (Figure 2).

### 2.3. Myocardial I/R Injury Model with Mouse Isolated Heart

The Langendorff retrograde heart perfusion system with isolated mouse heart was used to evaluate the myocardial function injury and recovery. Following anesthesia with sodium pentobarbital (60 mg/kg), hearts were excised and cannulated via the aorta. Perfusion was performed with oxygenated Krebs–Henseleit buffer (119 mM NaCl, 22 mM NaHCO_3_, 4.7 mM KCl, 1.2 mM KH_2_PO_4_, 1.2 mM MgSO_4_, 5.5/11 mM glucose, 2.5 mM CaCl_2_, 0.4/0.8 mM oleic acid, and 50 units/L Insulin, 37 °C, pH 7.4) containing glucose (5.5/11 mM) and oleic acid (0.4/0.8 mM). A balloon-tipped catheter was inserted into the left ventricle to maintain left ventricular end-diastolic pressure (LVEDP) at 8–10 mmHg. Hemodynamic parameters, including left ventricular developed pressure (LVDP), Rate-LV Pressure Product (RPP), and the maximum rate of left ventricular pressure rise (+dp/dt) and decline (−dp/dt), which are established indices of cardiac systolic and diastolic function, were continuously recorded using a BL-420F Biological Function Experiment System (Chengdu Taimeng Instrument, Sichuan, China). Global ischemia (25 min) and reperfusion (45 min) were induced via a three-way valve. At endpoint, hearts were collected for TTC staining and further analysis.

### 2.4. 2,3,5-Triphenyltetrazolium Chloride (TTC) Staining

After 45 min of reperfusion, hearts were promptly excised and rinsed with ice-cold physiological saline. The specimens were snap-frozen at −20 °C for 20 min, then sectioned transversely from apex to base into 1 mm thick slices. Tissue sections were treated with a 1% triphenyltetrazolium chloride (TTC) dissolved in PBS buffer at 37 °C in a light-proof environment for 20 min to distinguish viable (red) from infarcted (pale) myocardium. Subsequently, high-resolution digital imaging of the stained sections was carried out using a scanner. Quantification of infarct size was achieved through analysis with ImageJ software (NIH) (version 1.53), and the myocardial infarction area was expressed as a percentage of the total ventricular area (Infarction ratio = [Infarcted area/Total myocardial area] × 100%).

### 2.5. Quantification of Myocardial Succinate Accumulation by High-Performance Liquid Chromatography

During heart perfusion experiments, perfusate samples from each group were collected at three specific time points: 35 min after perfusion initiation, at the end of the ischemic phase, and 1 min after reperfusion. Subsequently, the samples were promptly treated with 10% perchloric acid, rapidly frozen in liquid nitrogen, and stored at −80 °C for later analysis. Following collection, the samples were centrifuged at 12,000× *g* to eliminate insoluble materials. Metabolites were separated using high-performance liquid chromatography on a C18 reversed-phase column with a 10 mmol/L KH_2_PO_4_ mobile phase (flow rate: 0.5 mL/min), with a 20-μL sample injection. Succinate was identified by monitoring absorbance at 210 nm using a diode array detector (Agilent 1260 Infinity II, Santa Clara, CA, USA).

### 2.6. Isolation of Myocardial Mitochondria

Cardiac mitochondria were isolated via a two-step centrifugation. Excised mouse hearts were rinsed in physiological saline and relaxed in pre-cooled relaxation buffer (5 mM HEPES, 5 mM EGTA, 100 mM KCl, pH 7.4) for 15 min. The tissue was minced and homogenized in ice-cold isolation buffer (5 mM HEPES, 1 mM EDTA, 250 mM sucrose, pH 7.4). The homogenate was centrifuged at 800× *g* for 10 min at 4 °C to remove debris, and the supernatant was further centrifuged at 12,000× *g* for 10 min at 4 °C. The mitochondrial pellet was resuspended in assay buffer (2 mM HEPES, 1 mM EGTA, 70 mM sucrose, 5 mM KH_2_PO_4_, 5 mM MgCl_2_·6H_2_O, 220 mM mannitol, pH 7.4), and protein concentration was determined using a BCA assay kit (Beyotime Biotechnology, Shanghai, China). All steps were performed at 4 °C unless otherwise noted.

### 2.7. Measurement of Myocardial Mitochondrial H_2_O_2_ Release

Mitochondrial H_2_O_2_ generation was assessed using the Amplex Red/HRP-coupled assay. The production of H_2_O_2_ was determined in isolated mouse heart mitochondria using malic acid/glutamic acid and succinic acid as electron donors, respectively. Mitochondrial suspensions (0.25 mg protein/mL in assay buffer) were incubated with reaction mixtures containing 10 μM Amplex Red, 1 U/mL horseradish peroxidase (HRP), and one of the following substrates: (1) 2.5 mM malic acid/5.0 mM glutamic acid, (2) 2.5 mM malic acid/5.0 mM glutamic acid + 4.0 μM rotenone, (3) 2.5 mM malic acid/5.0 mM glutamic acid + 1.0 μM nuciferine, (4) 2.5 mM malic acid/5.0 mM glutamic acid + 4.0 μM rotenone + 1.0 μM nuciferine, or (5) 5.0 mM succinic acid, (6) 5.0 mM succinic acid + 4.0 μM rotenone, (7) 5.0 mM succinic acid + 1.0 μM nuciferine, (8) 5.0 mM succinic acid + 4.0 μM rotenone + 1.0 μM nuciferine. Reactions commenced with the addition of mitochondrial after substrate and reagent pre-equilibration. Fluorescence was monitored at 3 min intervals over 15 min at an excitation wavelength of 545 nm and an emission wavelength of 590 nm using a Multi-Mode Detection platform (Molecular Devices, San Jose, CA, USA). H_2_O_2_ concentrations were determined using a standard curve prepared under the same assay conditions.

### 2.8. Complex I Activity, SDH Activity and NAD^+^/NADH Ratio Assay

Complex I was assessed using NADH-CoQ reductase Activity Assay Kit (Beijing Solarbio Science & Technology Co., Ltd., Beijing, China), according to the manufacturer’s instructions. In short, the activity of complex I, which catalyzes the oxidation of NADH to NAD^+^, is assayed by monitoring the decrease in absorbance at 340 nm associated with NADH consumption. SDH activity was assessed using an SDH test kit (Code A022, Nanjing Jiancheng Bioengineering Research Institute, Nanjing, China). The levels of NAD^+^/NADH ratio were determined by the NAD^+^/NADH Assay Kit with WST-8 (Beyotime Biotechnology, Shanghai, China).

### 2.9. Western Blotting Assay

Left ventricular apical myocardial tissues were collected, homogenized and lysed with RIPA buffer on ice for 30 min. The lysates were centrifuged at 4 °C and 12,000× *g* for 10 min. The supernatant was used to measure protein concentration via the BCA assay. Loading buffer was added, and the samples were boiled for 10 min to denature the proteins. Proteins were separated using sodium dodecyl sulfate polyacrylamide gel electrophoresis (SDS-PAGE) and transferred to polyvinylidene Fluoride (PVDF) membranes. These membranes were blocked for 2 h in 5% skimmed milk solution and then washed. Subsequently, they were incubated overnight at 4 °C with the following primary antibodies (all diluted at 1:1000): dynamin-related protein 1 (Drp1), mitochondrial fusion protein 2 (Mfn2), peroxisome proliferator-activated receptor gamma coactivator 1-alpha (PGC-1α), Sirt1, mitochondrial transcription factor A (TFAM), and glyceraldehyde-3-phosphate dehydrogenase (GAPDH). After washing, the membranes were incubated with a horseradish peroxidase-conjugated secondary antibody (1:10,000) for 2 h. Finally, the band densities were analyzed using enhanced chemiluminescence (ECL) and ImageJ software (version 1.53).

### 2.10. Molecular Docking and Molecular Dynamics Simulation Study

Molecular docking was performed using AutoDock Vina (version 1.1.2). The crystal structures of Complex I (PDB: 5XTD), SDH (PDB: 8GS8), and Sirt1 (PDB: 4I5I) were retrieved from the PDB database. The structure of nuciferine was obtained from PubChem (https://pubchem.ncbi.nlm.nih.gov/compound/Nuciferine (accessed on 27 March 2025), converted to 3D format, and energy-minimized. The docking grid was centered on the native ligand binding site of each protein. Docking poses were ranked by predicted binding energy, and the most favorable conformation for each target was selected for interaction analysis using PyMOL (Version 2.6, Schrödinger, LLC, New York, NY, USA).

All-atom molecular dynamics (MD) simulations were systematically performed to elucidate the binding mechanisms of nuciferine with succinate dehydrogenase, starting from the optimal docking poses. These simulations utilized the AmberTools 20 suite on the Yinfo Cloud Platform (https://www.yinfotek.com/ (accessed on 27 March 2025)). Each system was solvated in an OPC water box with 0.15 M NaCl. Each system was solvated in a truncated octahedral box of OPC water with a 10 Å buffer and neutralized with 0.15 M NaCl. The ff19SB force field was applied to the protein, while GAFF2 with AM1-BCC charges was used for nuciferine. Following energy minimization and stepwise equilibration under NVT and NPT ensembles, a 20 ns production run was conducted for each complex under NVT conditions. Trajectory analysis was performed using CPPTRAJ (version from AmberTools 22) to calculate the root mean square deviation (RMSD) and fluctuation (RMSF), hydrogen bond (H-Bond) occupancy, and radius of gyration (Rg). The stability of each system was assessed by monitoring the convergence of RMSD values over the simulation time course. Stable trajectory segments were subsequently used for detailed interaction analysis.

### 2.11. Cell Line and Cell Culture

The human cardiomyocyte cell line AC16 was purchased from the National Collection of Authenticated Cell Cultures (Shanghai, China). The cells were cultured in DMEM/F-12 supplemented with 10% fetal bovine serum (FBS, Gibco BRL, New York, NY, USA) and 1% (*v*/*v*) penicillin/streptomycin (Beyotime Biotech.) at 37 °C in an atmosphere containing 5% CO_2_.

### 2.12. Lipotoxicity Combined with Hypoxia–Reoxygenation Model

Cells were cultured to approximately 80% confluency, then washed with D-Hanks buffer, detached using trypsin, and collected by centrifugation. A single-cell suspension was prepared, and the cell density was adjusted to 0.7 × 10^4^ cells/mL before seeding into a 48-well plate. After cell adherence, these cells were treated with culture medium containing varying concentrations of oleic acid for 24 h. The plate was then transferred to an anoxic chamber and subjected to a gas mixture of 95% N_2_ and 5% CO_2_, pre-scrubbed through a 5% (*w*/*v*) sodium dithionite solution to ensure complete oxygen depletion. Hypoxic culture was maintained for 12 h, followed by 22 h of reoxygenation in a normoxic CO_2_ environment. Subsequent assays were performed following this hypoxia–reoxygenation protocol.

### 2.13. Cell Morphology Observation

Cells were plated in 48-well culture plates and maintained under standardized conditions. Upon completion of the experiment, cells were systematically processed and imaged using phase-contrast microscopy (inverted microscope configuration) to capture morphology.

### 2.14. Sulforhodamine B Assay

Cell proliferation was determined by the sulforhodamine B (SRB) assay [25]. Cells were seeded in a 48-well plate for predetermined durations. They were fixed with 125 µL of 50% (*v*/*v*) trichloroacetic acid (Sinopharm Reagent, Shanghai, China) for 60 min at 4 °C and washed five times using distilled water. After washing and fixation, cells were stained with 350 µL of 0.4% (*w*/*v*) SRB (Sigma-Aldrich, St. Louis, MO, USA) in 1% acetic acid for 60 min at room temperature. The wells were then rinsed with 1% acetate acid and air dried. The stain was dissolved in 500 µL of 10 mM Tris (pH 10.5), and absorbance was measured at 540 nm using a Multi-Mode Detection platform (Molecular Devices).

### 2.15. Lactate Dehydrogenase Release Assay

The release of cytoplasmic lactate dehydrogenase (LDH) serves as a marker of compromised cell membrane integrity, and thus, cell death [26]. Following treatment, the supernatants of AC16 cells were collected, and LDH levels were quantified using a commercial LDH assay kit (Beyotime), according to the manufacturer’s instructions. Absorbance was measured at 490 nm using a Multi-Mode Detection platform, and the relative LDH release was calculated as an indicator of cell damage.

### 2.16. Evaluation of AC16 Cell Apoptosis by AO/EB Staining and Flow Cytometry (FCM)

Acridine orange (AO) and ethidium bromide (EB) dual staining was employed to evaluate morphological changes indicative of apoptosis or necrosis. Cells were washed twice with PBS, stained with AO/EB (0.1 mg/mL), and observed under a fluorescence microscope at 200× magnification. Apoptosis quantification was conducted using a commercial Annexin V-FITC/PI Apoptosis Assay Kit (Beyotime Biotechnology, Shanghai, China). according to the manufacturer’s instructions. Harvested cells were dual-stained with Annexin V-FITC and propidium iodide (PI) in the dark for 10 min, followed by fluorescence-activated cell sorting (FACS) analysis using a Coulter flow cytometer (Beckman Coulter, Inc., Brea, CA, USA) Apoptotic populations were defined as Annexin V-FITC-positive/PI-negative (early apoptosis) and Annexin V-FITC/PI-double-positive (late apoptosis), with gating strategies validated against unstained and single-stained controls.

### 2.17. Change in Mitochondrial Membrane Potential (JC-1 Staining)

5,5′,6,6′-Tetrachloro-1,1′,3,3′-tetraethyl-imidacarbocyanine (JC-1) assesses changes in mitochondrial membrane potential (MMP) [27]. Cells were seeded in 48-well plates and cultured to the experimental endpoint. Post-treatment, cells were gently washed with phosphate-buffered saline (PBS) to remove media and debris. For MMP assessment, cells were stained with JC-1 dye (5 μg/mL) and incubated at 37 °C with 5% CO_2_ in the dark for 15 min. After incubation, cells were washed twice with PBS to remove excess dye. Fluorescence imaging was then performed using an inverted fluorescence microscope, capturing red (ex/em: 550/600 nm, J-aggregates) and green (ex/em: 485/535 nm, monomeric) emission signals.

### 2.18. Measurement of Intracellular and Mitochondrial ROS

To assess ROS production, 2,7-dichlorofuorescin diacetate (DCFH-DA) was employed using a ROS detection kit (Nanjing Jiancheng Bioengineering Research Institute, China). AC16 cells were seeded in 6-well plates for specified durations. Thereafter, cells were collected, washed thrice with PBS, and incubated with either 10 µM or 5 µM DCFH-DA for 30 min at 37 °C in the dark. Subsequently, cells were washed with PBS, detached using trypsin/EDTA, and the supernatant was discarded. The cell pellet was resuspended in PBS and analyzed via flow cytometry as per the manufacturer’s protocol. A hydrogen donor served as the positive control, while cell with or without the DCFH-DA acted as blank or negative controls. All experiments were performed in triplicate.

### 2.19. Statistical Analyses

Data were presented as mean ± standard error (SEM) from a minimum of three independent experiments. Statistical analyses utilized GraphPad Prism 9. An unpaired, two-tailed Student’s *t*-test was employed for two-group comparisons, one-way ANOVA with post hoc tests was used for multi-group analyses and two-way ANOVA with Tukey’s multiple comparisons test was applied for pairwise comparisons. A *p*-value < 0.05 was considered statistically significant.

## 3. Results

### 3.1. High Glucose Combined with High Oleic Acid Perfusion Aggravates Myocardial Ischemic/Reperfusion Injury in Isolated Mouse Heart

**Figure 3:**
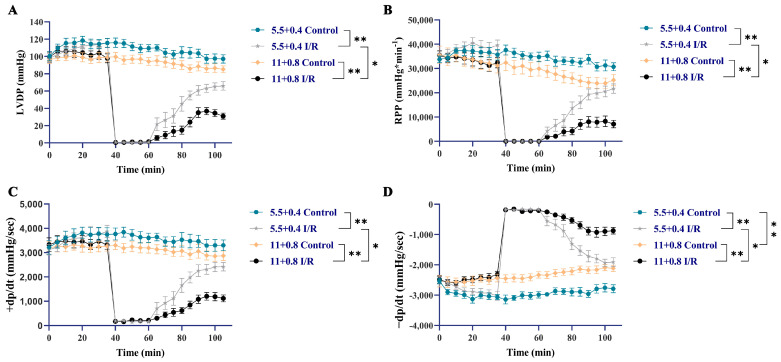
Development of an experimental model for myocardial ischemia–reperfusion injury aggravated by high glucose and fatty acids. (**A**–**D**) Measurements of LVDP, RPP and ±dp/dt for each heart group. Data are presented as mean ± SEM (*n* = 6). * *p* < 0.05, ** *p* < 0.01.

Utilizing the Langendorff isolated heart perfusion system (Radnoti 130102EZ, Radnoti LLC, Monrovia, CA, USA), we established a model of myocardial ischemia/reperfusion injury exacerbated by high glucose and fatty acid conditions to investigate the cardioprotective effects of nuciferine. Our findings indicated that high glucose and fatty acid conditions aggravated the deterioration of cardiac functional recovery after myocardial ischemia/reperfusion in mice (Figure 3A–D). Consistent with the established view that left ventricular diastolic dysfunction is a primary feature of diabetic heart disease [28], we observed that perfusion with a high-glucose and high oleic acid KH buffer (in the absence of I/R injury) significantly elevated -dp/dt in mouse hearts (Figure 3D). This result demonstrates impaired left ventricular diastolic function and aligns with the classic pathology of diabetic cardiomyopathy.

### 3.2. Nuciferine Attenuates High Glucose/Oleic Acid-Exacerbated Myocardial Ischemia/Reperfusion Injury

**Figure 4:**
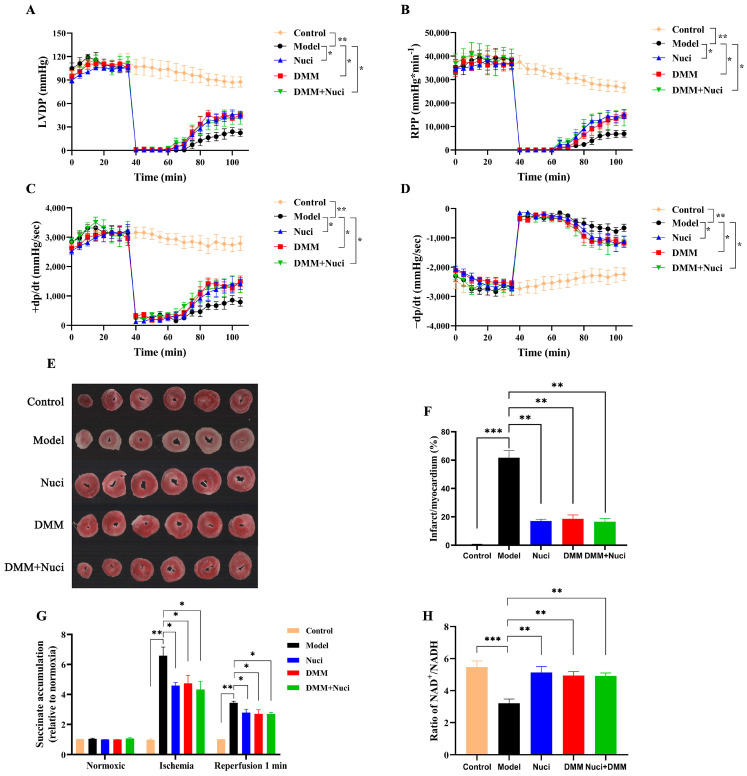

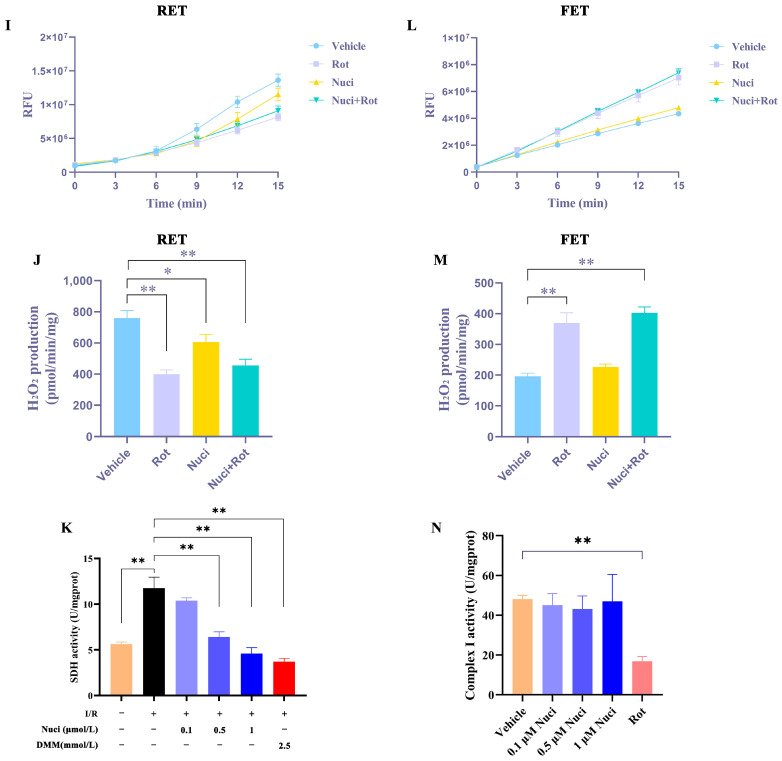
Nuciferine enhances cardiac function and mitigates lipotoxicity-aggravated myocardial I/R injury in mice. (**A**–**D**) Measurements of LVDP, RPP, and ±dp/dt for each group of hearts pre-ischemia, during ischemia and post-ischemia (*n* = 6). (**E**,**F**) The results of TTC staining and corresponding statistical analysis for each heart group (*n* = 3). (**G**) Quantification of succinate in cardiac perfusion fluid for each group (*n* = 3). (**H**) Influence of nuciferine or DMM on NAD^+^/NADH ratio (*n* = 3). (**I**,**J**) Assessment of RET-ROS production using succinate as the substrate, evaluating the impact of 1 µM nuciferine or 4 µM rotenone on succinate-induced H_2_O_2_ production by RET (*n* = 3). (**K**) Influence of nuciferine or DMM on SDH activity (*n* = 3). (**L**,**M**) Assessment of FET-ROS production using malate/glutamate as the substrate. (*n* = 3). (**N**) Influence of nuciferine on Complex I activity (*n* = 3). Data are presented as mean ± SEM. * *p* < 0.05, ** *p* < 0.01, *** *p* < 0.001.

Two-way ANOVA revealed no significant interaction between nuciferine and DMM treatments. Comparative analysis with I/R group, nuciferine significantly enhanced cardiac functional recovery and reduced myocardial damage during reperfusion, while DMM alone or combined with nuciferine exhibited similar protective effects (Figure 4A–D). As assessed by TTC staining, the extent of myocardial infarction was substantial in the I/R group. Conversely, treatment with nuciferine, DMM, or their combination (DMM + Nuci) resulted in a significant reduction in infarct size (Figure 4E,F).

LC-MS-based metabolomic profiling of murine brain, kidney, liver, and heart under in vivo ischemia has established the selective accumulation of the tricarboxylic acid (TCA) cycle intermediate succinate as a universal hallmark of ischemic stress. Demonstrating a consistent 3 to 19-fold increase across these metabolically diverse tissues, succinate stands as the singular mitochondrial metabolite universally elevated. This conserved accumulation is implicated in the extensive generation of ROS via the mechanism of RET at mitochondrial complex I [10]. To evaluate the contribution of succinate to the ROS formation post-myocardial ischemia, heart perfusate was collected at different intervals for quantitative analysis of succinate. Results showed a significant increase in succinate following ischemia. Conversely, treatment with nuciferine, DMM, or their combination markedly reduced succinate accumulation in the myocardial tissues at terminal stage of ischemia and 1 min after reperfusion (Figure 4G). Concurrently, nuciferine significantly elevated the NAD^+^/NADH ratio in myocardial tissue (Figure 4H). To investigate nuciferine’s role in mitochondrial ROS regulation, RET-ROS production was measured in isolated mouse heart mitochondria using succinate as the electron donor [19]. Exogenous succinate significantly enhanced the H_2_O_2_ production, which was notably reduced by both rotenone and nuciferine preincubation (Figure 4I,J). Cardiac tissue exhibited a significant elevation in SDH activity following I/R injury. This increase was concentration-dependently suppressed by nuciferine, with 1.0 μM nuciferine exhibiting a similar effect as the 2.5 mM DMM (Figure 4K). To further verify whether nuciferine exerts its protective effect via modulation of SDH activity, forward electron transport (FET)-dependent ROS production was assessed using glutamate/malate as electron donors; complex I activity was also measured. The results indicated that nuciferine did not significantly affect complex I activity (Figure 4L–N). Collectively, these findings demonstrate that nuciferine mitigates I/R injury under high-fat stress by modulating SDH activity, thus reducing RET-ROS production and preserving cardiac function, similar to the DMM.

### 3.3. Molecular Docking and Molecular Dynamics Assessment

**Figure 5:**
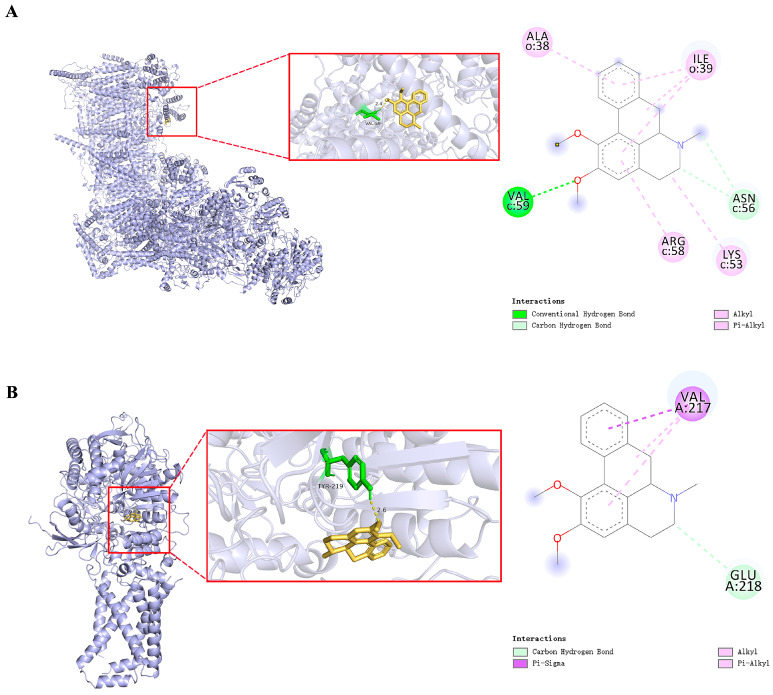

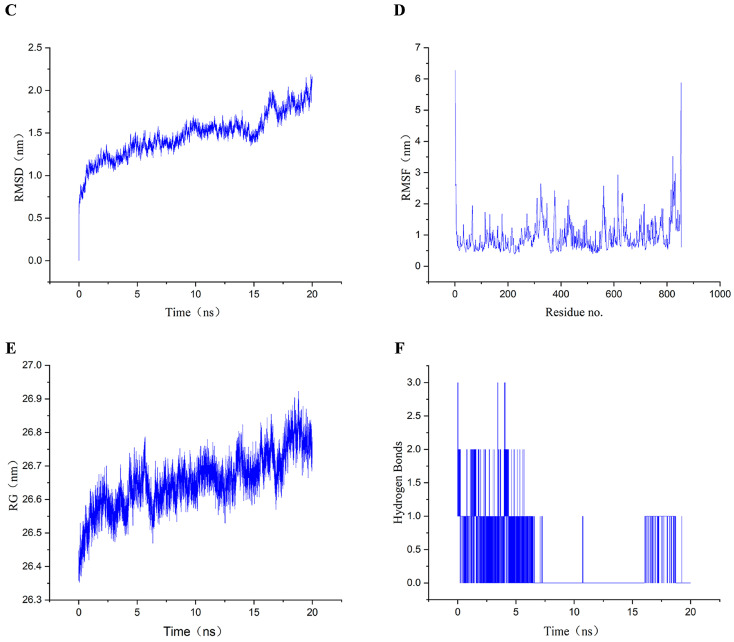
Visualization of nuciferine interconnecting with complex I and SDH. (**A**) Complex I. (**B**) SDH. (**C**) RMSD of protein backbone in the nuciferine-bound structure of SDH. (**D**) RMSF values of protein over time. (**E**) Rg values of proteins. (**F**) Number of hydrogen bonds formed between the nuciferine and SDH.

Docking energy quantifies the energy change when a small molecule interacts with a target protein, crucial for predicting binding modes and affinities in drug design and screening. It evaluates interactions between small molecules and proteins, with docking energy less than −4.25 kcal/mol suggesting binding capability, and less than −5.0 kcal/mol indicating strong binding [29,30]. Nine molecular docking calculations were conducted to determine the mean and standard deviation, revealing that nuciferine exhibits strong binding affinity toward both complex I and SDH, with consistent docking energies below −5.0 kcal/mol. Notably, the interaction with SDH was more favorable, as detailed in Table 1. PyMOL structural visualization confirmed nuciferine’s favorable binding within the active sites of these targets. These results underscore nuciferine’s potential as a modulator of SDH, supporting its therapeutic use in conditions related to mitochondrial dysfunction and oxidative stress (Figure 5A,B).

To validate the molecular docking results, we performed extensive molecular dynamics simulations to assess the stability and interaction dynamics of nuciferine-SDH complexes. Root mean square deviation analysis indicated exceptional complex stability, with fluctuations remaining within 1 Å throughout the simulation (Figure 5C and Figure S1), suggesting a highly stable binding mode between nuciferine and SDH. Root mean square Fluctuation measurements showed moderate residue flexibility (1–5 Å range), indicating optimal binding dynamics that preserve protein structural integrity while allowing necessary conformational adjustments (Figure 5D and Figure S2). Radius of gyration analysis confirmed structural compactness, with consistent values averaging 26 nm during the simulation (Figure 5E and Figure S3). This stability, without any unfolding events, highlighted the robustness of the nuciferine-SDH interaction. Hydrogen bond analysis revealed persistent intermolecular interactions, with at least one stable H-Bond maintained throughout (Figure 5F and Figure S4). These MD simulations provide compelling evidence for the formation of a highly stable nuciferine-SDH complex.

### 3.4. Nuciferine Regulates Mitochondrial Biogenesis and Maintains Fusion-Fission Homeostasis

**Figure 6:**
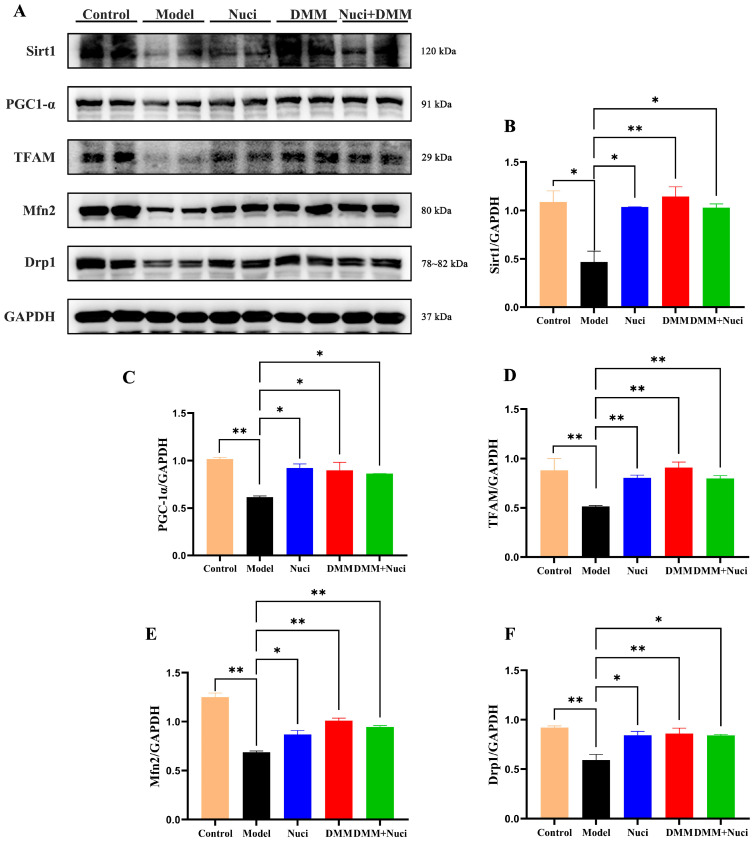
Effects of nuciferine on the expression of Sirt1, PGC-1α, TFAM, Mfn2, and Drp1 in ischemic/reperfusion-injured myocardial tissue. (**A**) Representative Western blot images of evaluated proteins. Quantitative analysis of the protein expression levels of (**B**) Sirt1, (**C**) PGC-1α, (**D**) TFAM, (**E**) Mfn2, and (**F**) Drp1. Data are presented as mean ± SEM (*n* = 3). * *p* < 0.05, ** *p* < 0.01.

The tightly regulated balance of mitochondrial fusion and fission—a fundamental process that preserves cellular metabolic homeostasis through quality control via mitophagy—is essential for eukaryotic cells. Western blot analysis was used to assess the expression of key proteins related to mitochondrial biogenesis and fusion-fission. As shown in Figure 6A–F, the I/R injured heart showed significant downregulation of Sirt1, PGC-1α, and TFAM in the myocardial tissue compared to the balanced perfusion group. Meanwhile, levels of the Mfn2 and Drp1 were markedly decreased. These results indicated that oleic acid exposure combined with I/R disrupts mitochondrial fusion-fission balance and biogenesis signaling in mouse heart. Pretreatment with nuciferine or DMM significantly restored these protein levels post-oleic acid stress and I/R. These findings suggested that nuciferine confers cardioprotection by improving mitochondrial biogenesis and maintaining fusion-fission homeostasis.

### 3.5. Oleic Acid Exacerbates H/R-Induced Cardiac Myocytes Injury

**Figure 7:**
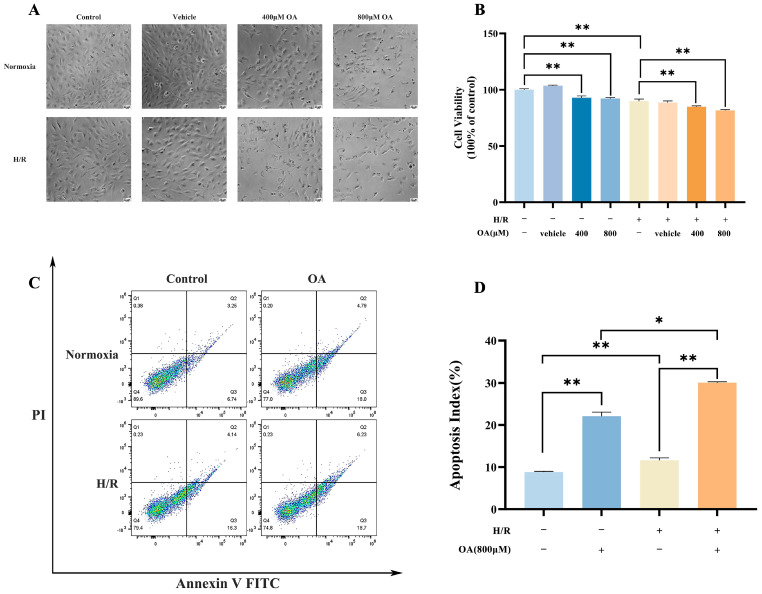
Oleic acid (OA) exacerbates H/R-induced myocardial cell injury. (**A**) Oleic acid combined with H/R alters the morphology of AC16 cardiomyocytes. (**B**) AC16 cells were treated with specified concentrations of oleic acid under H/R conditions, and cell viability was assessed using SRB assays. (**C**) Apoptotic cells were quantified using Annexin V/FITC-PI assay. In the flow cytometry scatter plot, normal cells appear in the lower left quadrant, naked nuclei or mechanically damaged cells in the upper left, late apoptotic cells in the upper right, and early apoptotic cells in the lower right. (**D**) Inter-group analysis examined the ratio of early and late apoptotic cells to total cells. Data above are expressed as the mean ± SEM from three independent experiments. * *p* < 0.05, ** *p* < 0.01.

To further identify the underlying mechanisms of nuciferine, the oleic acid combined with H/R induced AC16 cardiomyocytes damage to simulate the aggravated myocardial I/R injury caused by high glucose and lipid. Compared to normoxic controls, exposure to 400 μM or 800 μM oleic acid markedly inhibited cell proliferation and reduced cell density. Higher concentrations (800 μM) also caused notable morphological changes, such as cellular crumpling and fragmentation (Figure 7A). Similarly, H/R alone decreased cardiomyocyte viability. Combined treatment with oleic acid (400 μM or 800 μM) and H/R led to a significantly greater reduction in cell density and extensive cellular damage compared to H/R alone (Figure 7B). Thus, pre-incubation with 800 µM oleic acid and H/R was used to model exacerbated lipotoxicity in cardiomyocytes. To assess cellular apoptosis destiny, cells were stained with PI and Annexin V. Flow cytometry revealed that both oleic acid and H/R treatment increased cellular apoptosis rates in AC16 cardiomyocytes compared to controls, while their combination induced a more pronounced apoptotic response than either treatment alone, indicating that oleic acid pre-treatment exacerbates H/R-induced apoptosis (Figure 7C,D). These results suggested that pre-incubation with 800 µM oleic acid diminishes cardiomyocyte tolerance to subsequent H/R injury.

### 3.6. Nuciferine Alleviates AC16 Cardiomyocytes Damage Induced by Oleic Acid Combined with H/R

**Figure 8:**
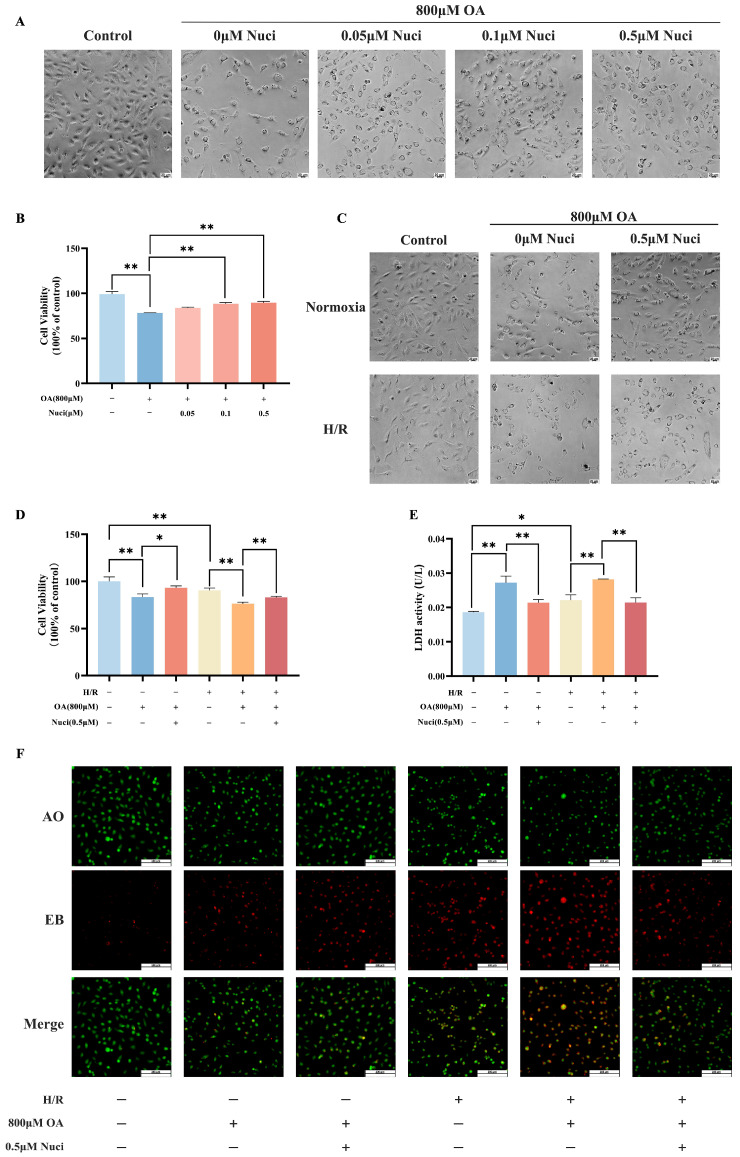

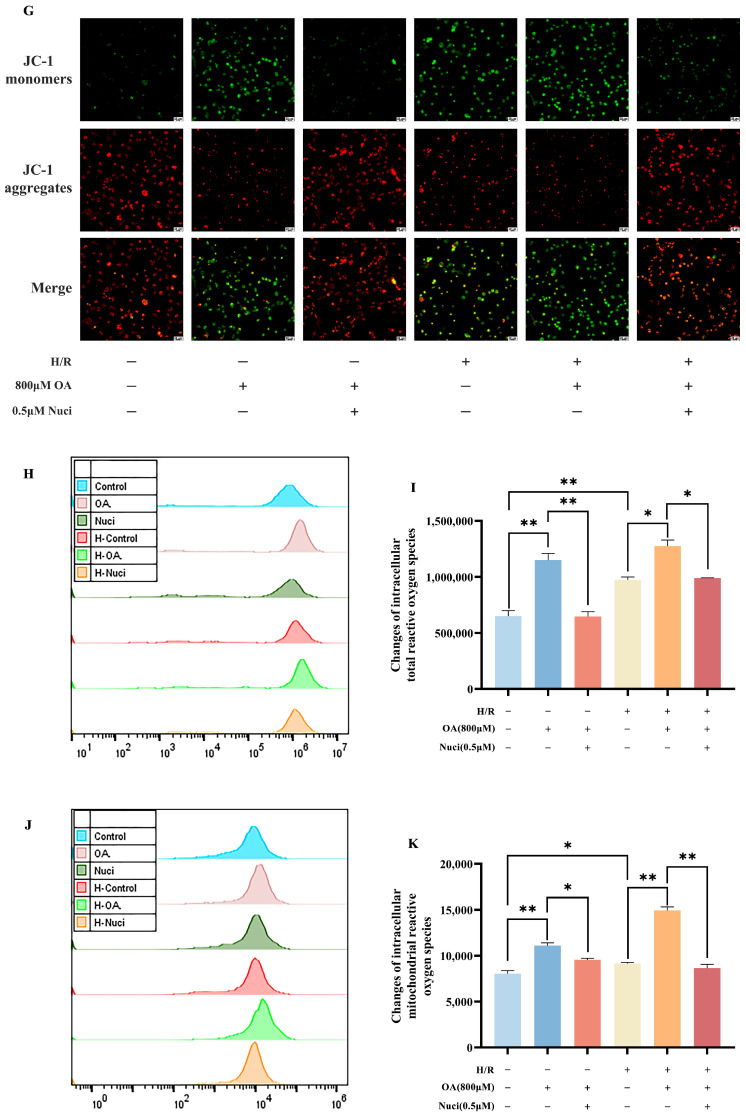
Nuciferine mitigates oleic acid and H/R-induced injury in AC16 cardiomyocytes. (**A**,**B**) Morphological and viability alterations in lipotoxicity-damaged AC16 cardiomyocytes post-nuciferine treatment. (**C**,**D**) Morphological and viability alterations in AC16 cardiomyocytes subjected to oleic acid and H/R injury following nuciferine administration. (**E**) Cytotoxicity assessed via LDH assays. (**F**) AO/EB dual staining under fluorescence microscopy: viable cells appeared uniformly green, apoptotic cells exhibited bright green nuclei due to chromatin condensation and nuclear fragmentation, and necrotic cells appeared bright orange. (**G**) JC-1 staining observed under fluorescence microscopy: red fluorescence represented intact mitochondrial membrane potential, while green fluorescence represented reduced potential. (**H**–**K**) Intracellular and mitochondrial ROS levels quantified by flow cytometry. Data above are expressed as the mean ± SEM from three independent experiments. * *p* < 0.05, ** *p* < 0.01.

The SRB assay assessed nuciferine’s effects on AC16 cardiomyocyte proliferation and viability. Co-treatment with nuciferine (0.05–0.5 µM) and 800 µM oleic acid for 48 h markedly attenuated oleic acid-induced reductions in cell density and viability, revealing a dose-dependent cardioprotective effect (Figure 8A,B). Nuciferine’s safety at concentrations ≤1.0 µM and efficacy at 0.5 µM informed its selection for further studies. Further results demonstrated that nuciferine notably mitigated the cytotoxic effects of oleic acid and H/R (Figure 8C,D). Pretreatment with nuciferine effectively countered the proliferation inhibition and increased cell density caused by oleic acid and H/R. Both oleic acid and H/R independently elevated extracellular LDH activity compared to controls (Figure 8E). The oleic acid combined with H/R treatment amplified LDH leakage beyond either intervention alone. Crucially, nuciferine treatment significantly reduced LDH release. These results collectively demonstrated that nuciferine enhances cell survival against oleic acid and H/R-induced injury.

The AO/EB double staining showed green homogeneous chromatin distributed in the nuclei of control cells. Conversely, oleic acid or H/R exposure significantly increased the red apoptotic cells stained by EB for their higher membrane permeability, with this effect further amplified when both were combined. Remarkably, nuciferine co-treatment markedly suppressed the apoptotic response induced by the combined oleic acid and H/R treatment (Figure 8F). To explore nuciferine’s protective effects on mitochondrial function, the JC-1 fluorescence probe was used to assess mitochondrial membrane potential (ΔΨm) in vitro. In healthy mitochondria, the high ΔΨm drives JC-1 into the mitochondria and forms aggregates emitting red fluorescence, while mitochondrial depolarization results in monomeric JC-1, which emits green fluorescence. Compared with untreated control group, oleic acid, H/R, or both of them in combination significantly increased green fluorescence and reduced red fluorescence intensity, indicating severe mitochondrial depolarization. Notably, nuciferine co-treatment substantially attenuated these pathological changes (Figure 8G). Next, the present study systematically examined the effects of the treatment on intracellular total and mitochondrial ROS generation using DCFH-DA and mitoSOX probes coupled with flow cytometry. Quantitative analysis showed that both oleic acid treatment and H/R injury independently increased intracellular total ROS (Figure 8H,I) and mitochondrial ROS (Figure 8J,K) levels compared to controls. Notably, their combination had a synergistic effect, further exacerbating ROS production beyond individual treatments. Pretreatment with nuciferine significantly reduced the intracellular total ROS and mitochondrial ROS accumulation induced by oleic acid alone or its combination with H/R. These findings suggested that nuciferine ameliorates oxidative stress and apoptosis under oleic acid and H/R conditions by protecting mitochondria.

### 3.7. Dimethyl Malonate Reverses AC16 Cardiomyocytes Damage Induced by Oleic Acid and H/R

**Figure 9:**
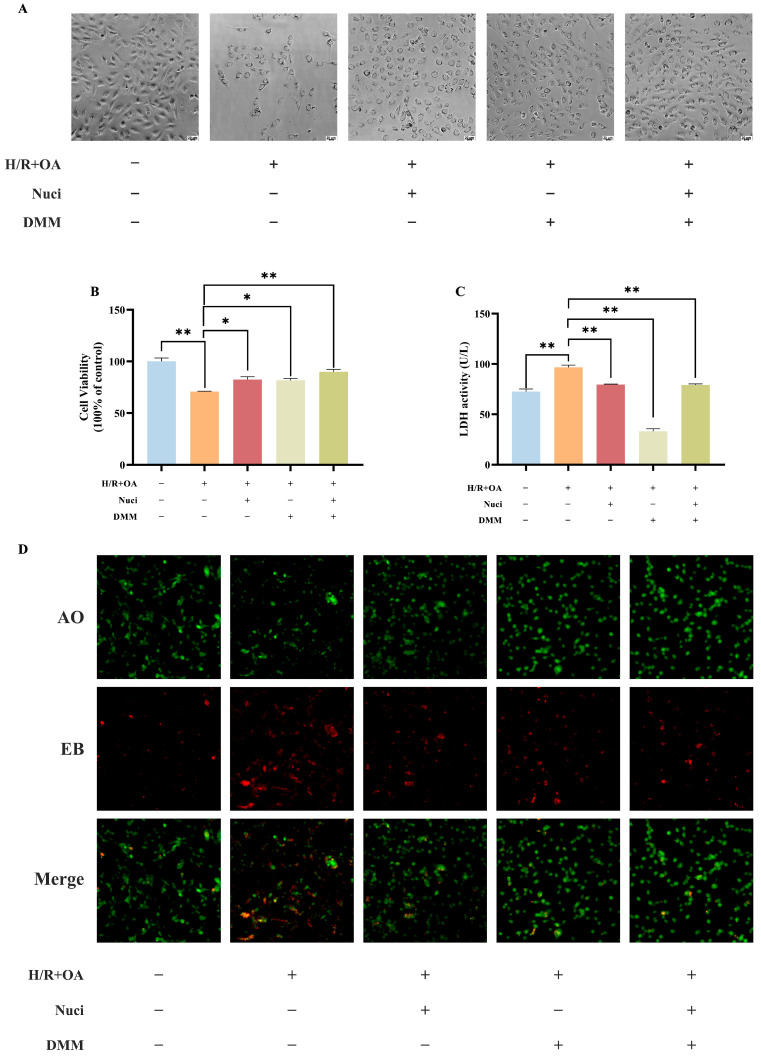

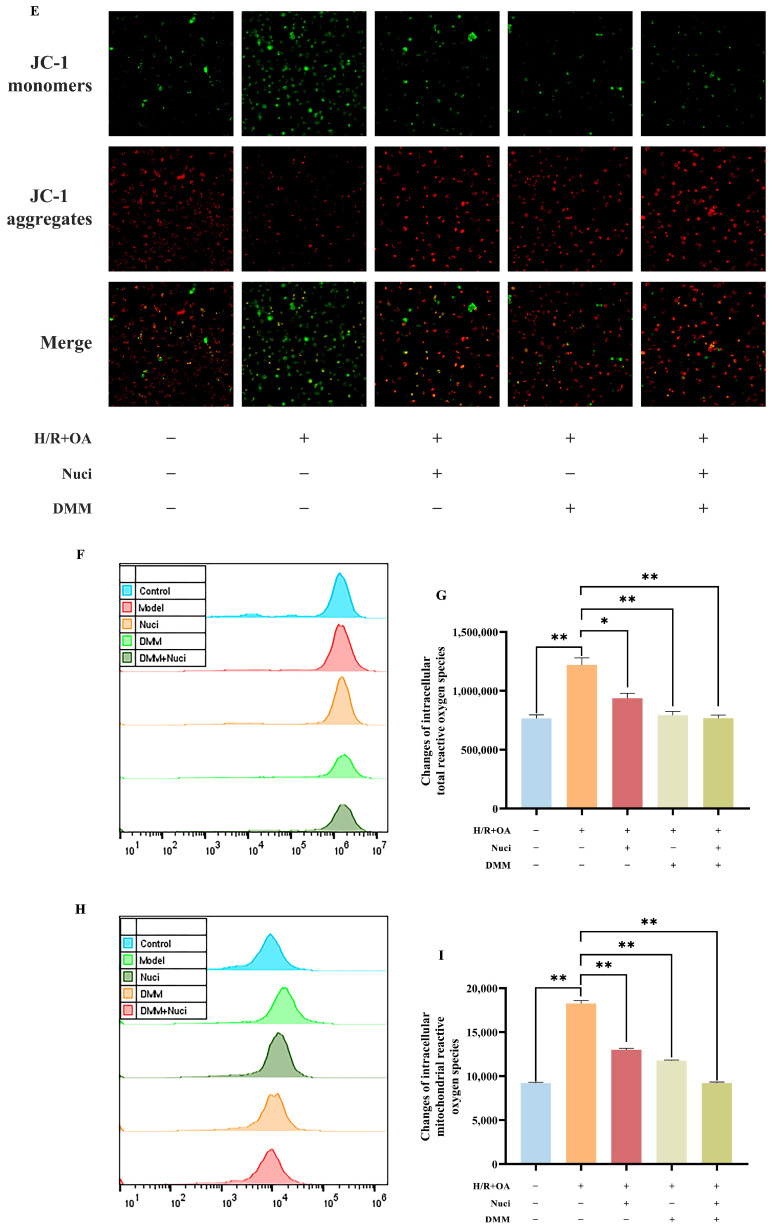
Amelioration of oleic acid and H/R-induced injury in AC16 cardiomyocytes by DMM or DMM with nuciferine. (**A**–**C**) The change in morphological, viability and LDH release alterations in AC16 cardiomyocytes. (**D**) AO/EB dual staining under fluorescence microscopy. (**E**) JC-1 staining observed under fluorescence microscopy. (**F**–**I**) Intracellular and mitochondrial ROS levels quantified by flow cytometry. Data above are expressed as the mean ± SEM from three independent experiments. * *p* < 0.05, ** *p* < 0.01.

This study explored the effect of dimethyl malonate (DMM), an SDH inhibitor, on oleic acid and H/R-induced injury in AC16 myocardial cells to clarify nuciferine’s mechanism of action. Pre-incubation with nuciferine (0.5 µM) or DMM (2.5 mM) significantly countered the suppression of cellular proliferation caused by oleic acid and H/R, with combined treatment showing improved activity but no synergy. Both agents, alone or together, significantly attenuated cell damage and reduced LDH release (Figure 9A–C). Acridine orange/ethidium bromide (AO/EB) staining confirmed a substantial increase in viable cells and a reduction in apoptotic rates following treatment with DMM or DMM with nuciferine (Figure 9D). Similarly, the protective effect of these treatments on mitochondrial integrity was confirmed by their ability to mitigate the loss of mitochondrial membrane potential (ΔΨm), evidenced by reduced green and increased red fluorescence (Figure 9E). Flow cytometry showed that both treatments reduced intracellular total ROS and mitochondrial ROS production (Figure 9F–I). The above results suggested that nuciferine’s cardioprotection may partly resemble SDH inhibition, potentially reducing pathological succinate accumulation during ischemic stress.

### 3.8. Inhibition of Sirt1 Attenuates the Protective Effect of Nuciferine on Cardiomyocyte Injury Induced by Oleic Acid Combined with H/R

**Figure 10:**
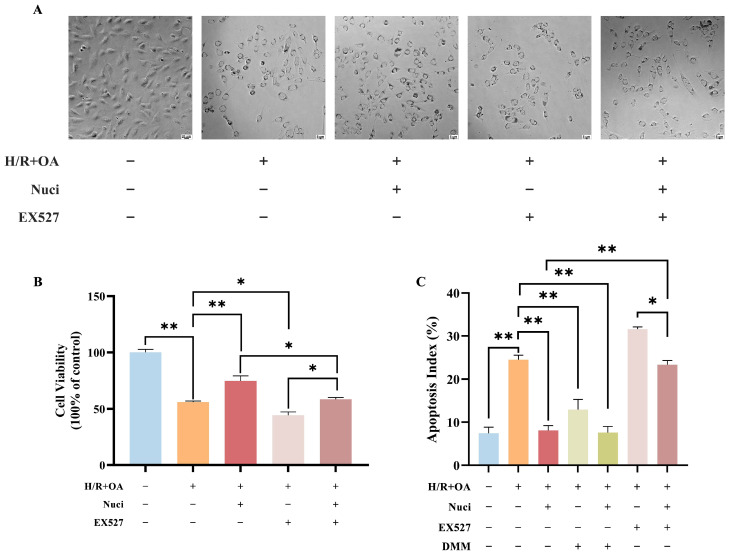

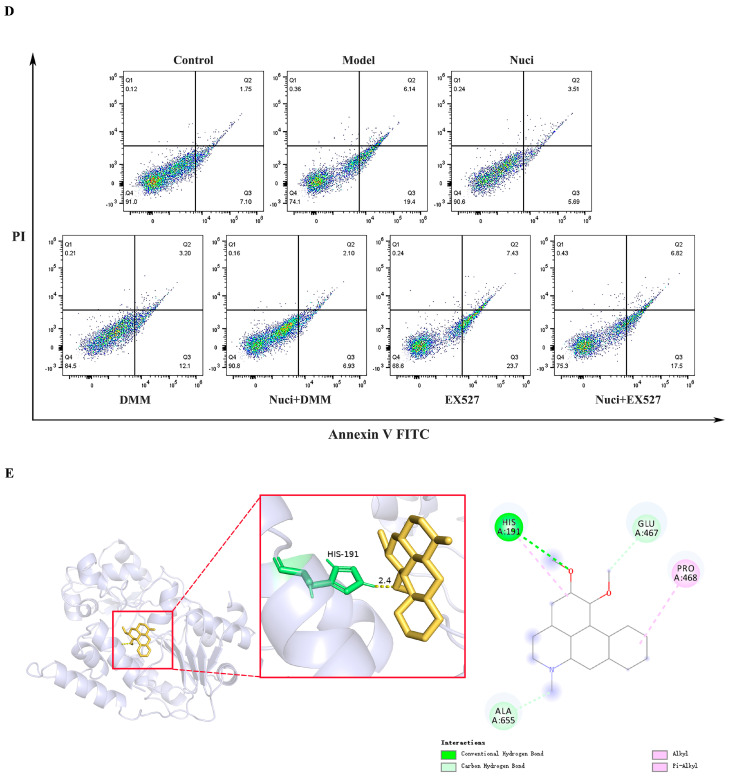
EX527 diminishes the protective effect of nuciferine in AC16 cardiomyocytes. (**A**,**B**) Morphological and viability changes in AC16 cardiomyocytes with oleic acid and H/R injury after treatment with 10 µM EX527 and/or nuciferine. (**C**) Inter-group analysis examined the ratio of early and late apoptotic cells to total cells. (**D**) Apoptotic cells were quantified using Annexin V/FITC-PI assay. (**E**) Visualization of nuciferine interconnecting with Sirt1. Data above are expressed as the mean ± SEM from three independent experiments. * *p* < 0.05, ** *p* < 0.01.

The Sirt1 inhibitor EX527 was employed to explore the relationship between nuciferine’s effects and Sirt1. Phase-contrast microscopy and SRB assay results indicated that EX527 intensified the cytotoxic effects induced by oleic acid and H/R, further reducing cell density and proliferation. Additionally, EX527 co-treatment nullified nuciferine’s cytoprotective effects, leading to significantly decreased cell density and proliferation compared to nuciferine treatment alone (Figure 10A,B). Annexin V/PI double staining demonstrated that nuciferine pretreatment markedly attenuated oleic acid and H/R-induced apoptosis in AC16 cardiomyocytes. This anti-apoptotic effect was notably diminished by EX527 co-treatment, as evidenced by a significant increase in apoptosis compared to nuciferine treatment alone. The experiments indicated that DMM, alone or with nuciferine, also reduced cardiomyocyte apoptosis and exhibited protective effects (Figure 10C,D). These findings established that Sirt1 activation is crucial for nuciferine’s anti-apoptotic activity against oleic acid and H/R injury. Molecular docking analysis demonstrated a favorable binding affinity between Sirt1 and nuciferine, with a binding energy of −6.1 kcal/mol. This stable interaction was structurally confirmed via PyMOL visualization, as shown in Figure 10E.

## 4. Discussion

The heart, a high-energy-consuming organ, primarily depends on long-chain fatty acid oxidation for its continuous contractile activity. However, excessive fatty acid intake and lipid metabolism modulating impairment, such as in type 2 diabetes mellitus (T2DM), can impair cardiac function and reduce its tolerance threshold for stress-induced damage [31]. Given nuciferine’s lipid-lowering and antioxidant properties [32], we explored its potential to protect against lipotoxicity-aggravated I/R injury. Hearts under high-energy density displayed significantly worse functional recovery and larger infarct size following I/R than those under low-energy perfusion. These results demonstrate the ability of nuciferine to attenuate myocardial injury under metabolically challenged conditions.

Mitochondrial ROS production is a crucial early factor in I/R injury, typically seen as a non-specific result of dysfunctional respiratory chain interactions with oxygen during reperfusion [33]. Recent evidence indicates that succinate accumulation during ischemia serves as a key metabolic driver of ROS production upon reperfusion. Upon reperfusion, succinate reduces CoQ to CoQH_2_ via SDH. CoenzymeQH_2_, with its high reduction potential, transfers electrons back to complex I through reverse electron transport, reducing O_2_ to ROS with a single electron. In this pathway, SDH serves as the primary driver for initiating electron flow into the quinone pool, while complex I acts as the major site for superoxide generation [34,35]. Together, they constitute two indispensable and synergistic components of the succinate-driven RET-ROS burst. This process is considered the primary cause of oxidative stress injury in myocardial tissue following I/R [36] and provides direct rationale for our subsequent focus on the functional analysis of complex I and SDH rather than screening the entire respiratory chain. In this study, we first confirmed that exogenous succinate markedly induced H_2_O_2_ generation in isolated cardiac mitochondria, an effect that was suppressed by the Complex I inhibitor rotenone. Notably, nuciferine produced a similar inhibitory effect, suggesting its action upstream of RET. Our experimental results similarly confirmed that ischemia significantly elevates succinate levels in cardiac tissue. Pretreatment with nuciferine or DMM notably reduced the accumulation of succinate. Importantly, the specific succinate dehydrogenase inhibitor DMM produced protective effects almost indistinguishable from those of nuciferine, with no significant additive or synergistic benefit observed upon co-administration. Recent studies suggest that DMM maintains tissue and mitochondrial functions following ischemia–reperfusion by inhibiting succinate dehydrogenase activity, thereby reducing succinate accumulation [37,38]. To further investigate whether nuciferine directly interferes with SDH activity, assays were conducted on mouse myocardial tissue. Succinate dehydrogenase (SDH) activity was notably higher in I/R-injured hearts compared to controls. Nuciferine attenuated SDH activity in a concentration-dependent manner but had no significant effect on complex I activity. Molecular docking and dynamics simulations suggested that nuciferine bound significantly to SDH. Collectively, these data support the conclusion that SDH inhibition is a principal mechanism through which nuciferine exerts cardioprotection, a function that appears largely analogous to that of the established SDH inhibitor DMM.

To further elucidate the protective mechanisms of nuciferine against myocardial I/R injury, we examined its impact on key regulators of mitochondrial homeostasis. Western blot analysis revealed that nuciferine significantly upregulated the expression of Sirt1, PGC-1α, TFAM, Mfn2, and Drp1 under lipotoxic and I/R conditions. These observations suggest that the cardioprotection conferred by nuciferine is associated with the activation of Sirt1, which subsequently coordinates mitochondrial biogenesis and dynamics [39]. The activity of Sirt1, a central NAD^+^-dependent deacetylase, is critically dependent on the intracellular NAD^+^/NADH ratio [20]. Our findings indicate that nuciferine suppresses SDH activity, thereby inhibiting RET and subsequently alleviating NAD^+^ depletion. RET can also cause NAD^+^/NADH imbalances, directly affecting Sirt1 activity. By preserving cellular NAD^+^ availability, nuciferine likely establishes a metabolic milieu favorable for Sirt1 activation. Once activated, Sirt1 can deacetylate and upregulate its key transcriptional coactivator PGC-1α, thereby enhancing the expression of downstream effectors such as TFAM to promote mitochondrial biogenesis. Concurrently, Sirt1-mediated regulation of proteins, including Mfn2 and Drp1, may help restore the balance between mitochondrial fusion and fission. Collectively, nuciferine alleviates myocardial injury by activating Sirt1-mediated mitochondrial biogenesis and restoring the homeostasis of mitochondrial fusion and fission.

Consistent with reports that saturated fatty acids enhanced fatty acid oxidation, induced cardiomyocyte hypoxia, and led to pathological succinate accumulation [40], we employed an AC16 cardiomyocyte model of oleate-induced lipotoxicity combined with H/R to complement our ex vivo findings. Consistent with isolated heart perfusion results, nuciferine mitigated oleic acid and H/R induced injury in AC16 cardiomyocytes, significantly decreased apoptosis, restored mitochondrial membrane potential, and reduced total and mitochondrial ROS. The SDH inhibitor DMM reproduced these cytoprotective effects, and co-administration with nuciferine provided no additive benefit, further supporting SDH inhibition as a primary mechanism for a major part of nuciferine’s protection. Clinical myocardial infarction often strikes without warning. Competitive inhibition of SDH has emerged as a promising strategy to suppress pathological RET during the clinically actionable therapeutic window. However, the successful translation of SDH inhibition critically depends on favorable pharmacokinetic properties. For instance, it has been reported that DMM failed to exert cardioprotection when given only at reperfusion in a murine infarction model, likely because its slow hydrolysis prevented effective competition with succinate oxidation during the narrow early therapeutic window [41]. In contrast, the natural compound nuciferine has shown promise, demonstrating significant cardioprotection against ischemia–reperfusion injury in vivo [42,43]. Notably, molecular docking revealed that nuciferine strongly bound to Sirt1, and the Sirt1 inhibitor EX527 markedly diminished nuciferine’s anti-apoptotic effect in AC16 cardiomyocytes. This indicates that Sirt1 pathway activation may independently contribute to nuciferine’s efficacy, a feature that could distinguish it from a pure SDH inhibitor.

Nevertheless, this study has limitations. While we have demonstrated that nuciferine attenuates myocardial I/R injury under metabolic stress by modulating SDH-mediated RET and activating Sirt1, the evidence remains constrained to ex vivo and cellular models. Consequently, the precise mechanisms underlying nuciferine’s action remain incompletely defined, spanning from its specific binding interaction with succinate dehydrogenase subunits to its potential engagement with other signaling pathways. These aspects necessitate clarification at the molecular and structural levels. Furthermore, this study only measured the activities of mitochondrial complexes I and SDH. To more systematically evaluate the impact of nuciferine on mitochondrial function, future research should supplement these measurements with assays of complexes III-V. Importantly, the translational relevance of these mechanisms remains to be validated in physiologically relevant in vivo systems, particularly in established diabetic models such as streptozotocin (STZ)/high-fat diet (HFD)-induced or db/db mice. These models recapitulate the systemic metabolic dysregulation and chronic cardiovascular pathology that mirror the complexity of clinical disease, thereby offering a more comprehensive view of its regulatory network in energy metabolism. Ultimately, key pharmacological properties of nuciferine, including its pharmacokinetics, optimal dosing, and long-term safety, remain to be characterized. Future work should address these gaps and position nuciferine within the broader context of mitochondrial-targeted therapies.

## 5. Conclusions

In summary, our results indicate that nuciferine exerts cardioprotective effects through a dual mechanism. This involves suppressing mitochondrial complex II reverse electron transport by modulating succinate metabolism, thereby inhibiting mitochondrial ROS generation and oxidative stress. Concurrently, nuciferine activates the Sirt1 pathway, reducing apoptosis and enhancing mitochondrial biogenesis. These findings provide new mechanistic insights and experimental evidence supporting nuciferine as a multi-target intervention strategy against cardiovascular injury exacerbated by metabolic stress.

## Figures and Tables

**Table 1:** Docking binding energy between complex I/SDH and nuciferine.

Subunit	Binding Affinity (kcal/mol)	Subunit	Binding Affinity (kcal/mol)
complex I	−8.0	SDH	−8.3
	−7.3		−8.3
	−7.1		−8.3
	−7.0		−8.3
	−7.0		−8.3
	−7.0		−8.2
	−6.8		−8.2
	−6.8		−8.2
	−6.3		−8.2
Mean ± SD	−7.14 ± 0.47	Mean ± SD	−8.26 ± 0.05

## Data Availability

The data that support the findings of this study are available from the corresponding author upon reasonable request. The data are not publicly available because they are stored on a personal server with limited storage capacity; therefore, they cannot be kept permanently available online.

## Supplementary Materials

The following supporting information can be downloaded at: https://www.mdpi.com/article/10.3390/nu18030425/s1, Figure S1: RMSD of protein backbone in the nuciferine bound structure of SDH. Root mean square deviation (RMSD) is a key metric for evaluating the stability of structure of SDH. A flatter RMSD curve indicates greater complex stability. Figure S2: RMSF values of protein over time. Root mean square fluctuation (RMSF) indicates the degree of fluctuation of amino acid residues during the simulation. Higher RMSF values correspond to greater residue mobility, whereas lower values reflect more restrained movement. Figure S3: Rg values of proteins. The radius of gyration (Rg) is a physical parameter describing the compactness of a protein structure. Smaller Rg values indicate a more compact and stable protein conformation. Figure S4: Number of hydrogen bonds formed between the nuciferine and SDH. The number of hydrogen bonds between the protein and the compound showed minimal fluctuation, remaining stable at 0 to 1 throug.

## Abbreviations

The following abbreviations are used in this manuscript:

AC16	human cardiomyocyte cells
AO	acridine orange
AMPK	5′ AMP-activated protein kinase
CoQ	coenzyme Q
CVDs	cardiovascular diseases
DMM	dimethyl malonate
Drp1	dynamin-related protein 1
EB	ethidium bromide
ECL	enhanced chemiluminescence
EX527	selisistat
FACS	fluorescence-activated cell sorting
FAD	flavin adenine dinucleotide
FCM	Flow Cytometry
FET	forward electron transport
FFAs	free fatty acids
FMN	flavin mononucleotide
GAPDH	glyceraldehyde-3-phosphate dehydrogenase
HFD	high-fat diet
H/R	hypoxia/reoxygenation
HRP	horseradish peroxidase
I/R	ischemia–reperfusion
KH	Krebs–Henseleit buffer
LDH	lactate dehydrogenase
LVEDP	left ventricular end-diastolic pressure
LVDP	left ventricular developed pressure
MD	molecular dynamics
Mfn2	mitochondrial fusion proteins 2
MI/R	myocardial ischemia/reperfusion
MMP	mitochondrial membrane potential
PDA	photodiode array
PGC-1α	peroxisome proliferator-activated receptor gamma coactivator 1-alpha
PI	propidium iodide
PVDF	polyvinylidene fluoride
RET	reverse electron transport
Rg	radius of gyration
RMSD	root mean square deviation
RMSF	root mean square fluctuation
ROS	reactive oxygen species
RPP	rate-LV pressure product
SDS-PAGE	sodium dodecyl sulfate polyacrylamide gel electrophoresis
Sirt1	sirtuin 1
SRB	sulforhodamine B
STZ	streptozotocin
TCA	tricarboxylic acid
TFAM	mitochondrial transcription factor A
TTC	2,3,5-Triphenyltetrazolium chloride
T2DM	type 2 diabetes mellitus
