# Supplementary material for: Nuciferine Ameliorates Lipotoxicity-Mediated Myocardial Ischemia–Reperfusion Injury by Reducing Reverse Electron Transfer Mediated Oxidative Stress
Source: Nutrients. 2026 Jan 27;18(3):425. doi: 10.3390/nu18030425 (PMC12899547; doi:10.3390/nu18030425)
Supplement: Supplementary file 1 [file nutrients-18-00425-s001.zip › nutrients-4092926-supplementary.pdf]

Supplemental Materials:

Nuciferine Ameliorates Lipotoxicity-Mediated Myocardial Ischemia–Reperfusion Injury by Reducing Reverse Electron Transfer Mediated Oxidative Stress

Man Wang [1], Xiaobing Shi [2], Yufeng Zhou [1], Jianhui Feng [1], Yining Diao [2], Gang Li [1], Zhenhua Wang [2,*] and Chengjun Ma [1,*]

1 Center for Mitochondria and Healthy Aging, School of Life Sciences, Yantai University, Yantai 264005, China; wangman202302@163.com (M.W.); m17865562177@163.com (Y.Z.); jhfeng0122@163.com (J.F.); ligang@ytu.edu.cn (G.L.)
2 Xinjiang Production and Construction Corps Key Laboratory of Protection and Utilization of Biological Resources in Tarim Basin, College of Life Science, Tarim University, Alar 843300, China; 15966463268@163.com (X.S.); addiaoyining@163.com (Y.D.)
* Correspondence: shkshysh@taru.edu.cn (Z.W.); skymcj@ytu.edu.cn (C.M.)

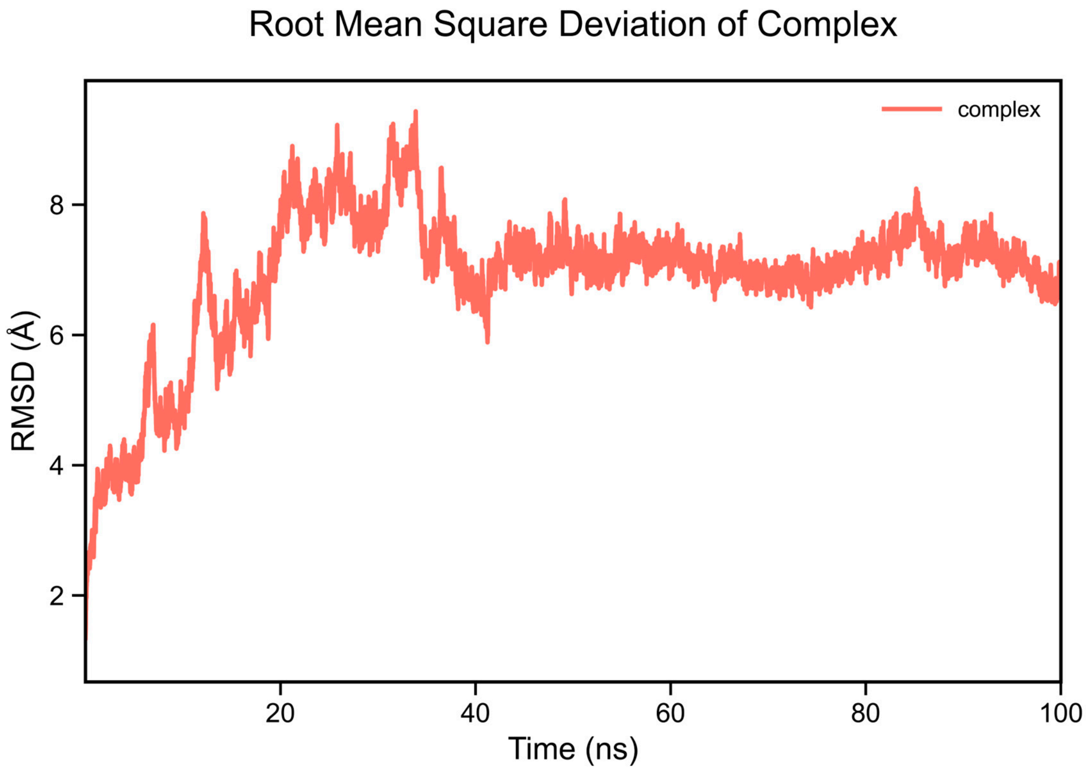

Figure S1. RMSD of protein backbone in the nuciferine bound structure of SDH. Root mean square deviation (RMSD) is a key metric for evaluating the stability of structure of SDH. A flatter RMSD curve indicates greater complex stability.

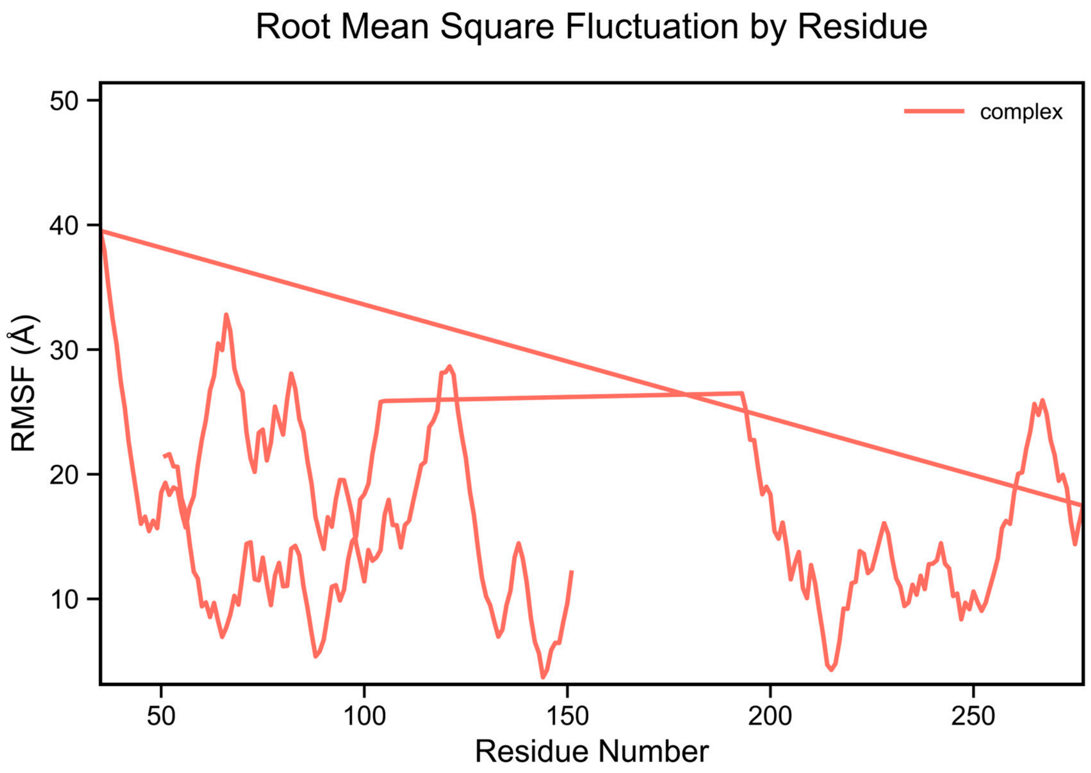

Figure S2. RMSF values of protein over time. Root mean square fluctuation (RMSF) indicates the degree of fluctuation of amino acid residues during the simulation. Higher RMSF values correspond to greater residue mobility, whereas lower values reflect more restrained movement.

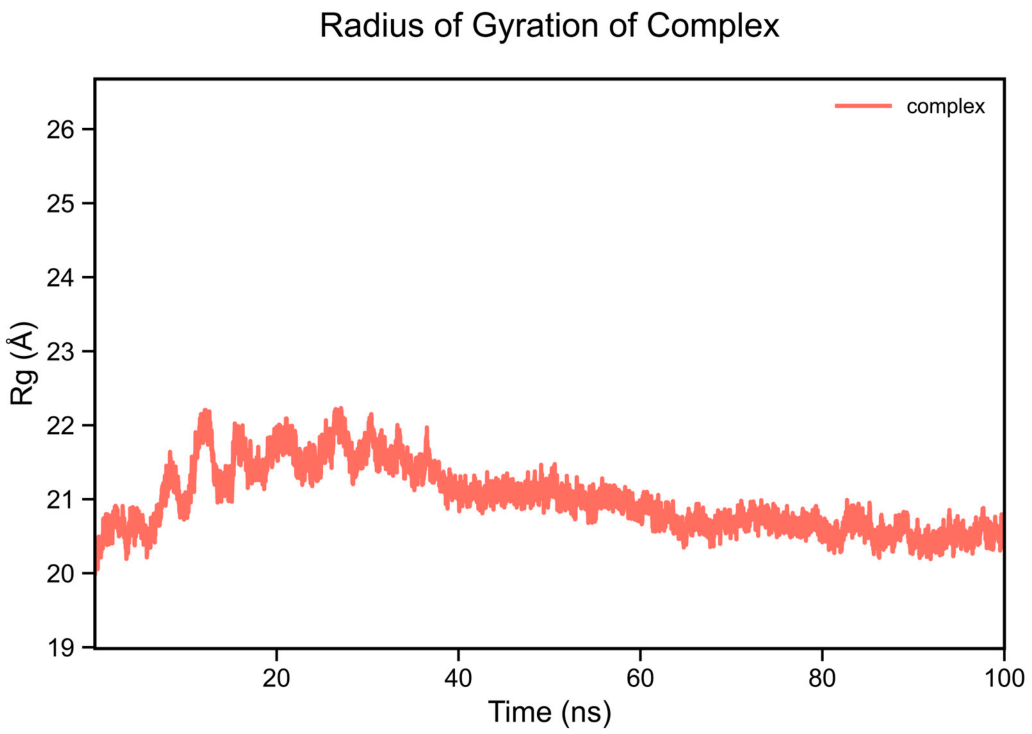

Figure S3. Rg values of proteins.The radius of gyration (Rg) is a physical parameter describing the compactness of a protein structure. Smaller Rg values indicate a more compact and stable protein conformation.

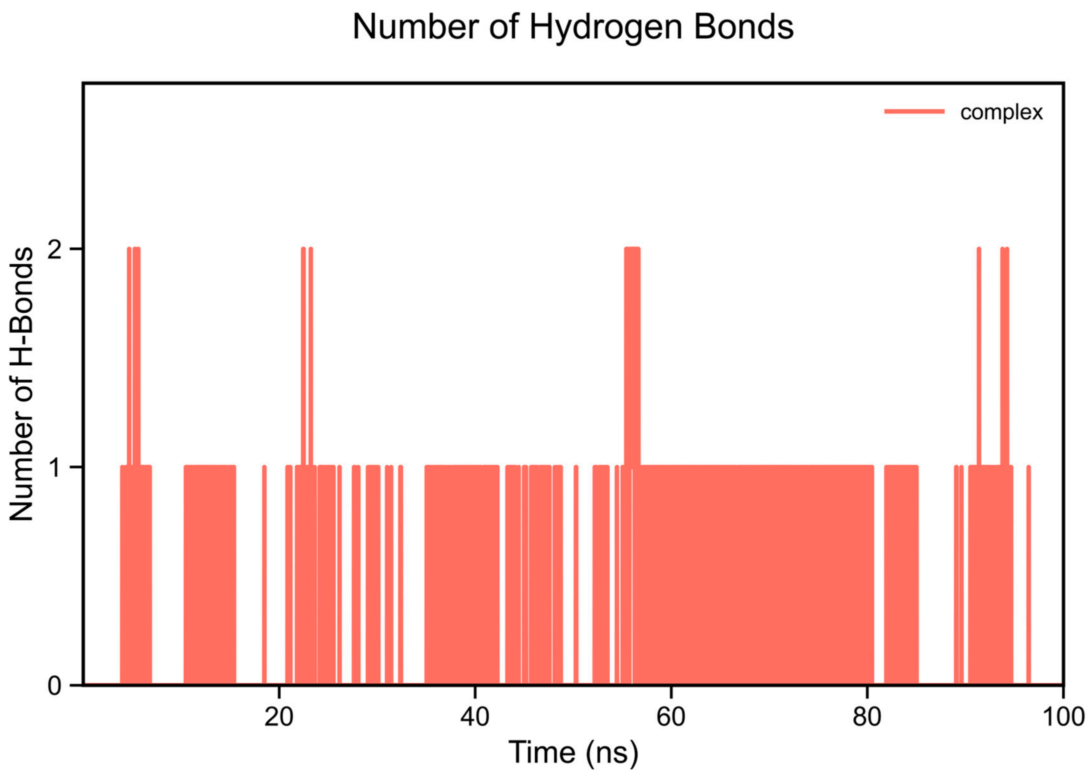

Figure S4. Number of hydrogen bonds formed between the nuciferine and SDH. The number of hydrogen bonds between the protein and the compound showed minimal fluctuation, remaining stable at 0 to 1 throughout the simulation.
